# The role of representation in participatory settings of health research in Germany: protocol for a scoping review

**DOI:** 10.1186/s40900-025-00736-w

**Published:** 2025-07-03

**Authors:** Tim Holetzek, Anke Desch, Corinna Klingler, Hester van de Bovenkamp, Christine Holmberg

**Affiliations:** 1 Institute of Social Medicine and Epidemiology, Brandenburg Medical School Theodor Fontane, Hochstraße 15, 14770 Brandenburg/Havel, Germany; 2 Brandenburg Medical School Theodor Fontane, Evidence Based Practice in Brandenburg – A JBI Affiliated Group, Brandenburg/Havel, Germany; 3 Institute of Ethics, History and Theory of Medicine, Ludwig-Maximilians-Universität München, Munich, Germany; 4 Erasmus School of Health Policy & Management, Erasmus University Rotterdam, Rotterdam, The Netherlands; 5 Faculty of Health Sciences Brandenburg, Brandenburg Medical School Theodor Fontane, Brandenburg/Havel, Germany

**Keywords:** Representation, Representativeness, Patient and public involvement, Health research, Germany

## Abstract

**Background:**

Participatory health research (PHR) has gained growing importance as an approach to include the perspectives of patients and communities in health research. While it is widely assumed that PHR contributes to more democratic and needs-based research, the conceptual foundations of how participation and representation are understood often remain vague – particularly in the German research context. This scoping review explores how representation is conceptualized and operationalized in participatory health-related research in Germany.

**Methods:**

The review follows the JBI methodology for scoping reviews and is reported in accordance with the PRISMA-ScR guidelines. A systematic search will be conducted across four databases (PubMed, CINAHL, PsycINFO, Scopus), supplemented by manual searches in the German PartNet archive and reference tracking. Eligible studies must report on empirical, participatory health research conducted in collaboration with a German research institution. Documents will be screened and analyzed using qualitative content analysis in MAXQDA, following both deductive and inductive coding strategies. Representation will be analyzed along two conceptual lines: representation as a responsive relationship and externally ascribed representation.

**Expected outcomes:**

The review will map existing understandings and practices of representation in German PHR. Findings will support future methodological and normative debates around participation in health research and provide a foundation for international comparison.

**Supplementary Information:**

The online version contains supplementary material available at 10.1186/s40900-025-00736-w.

## Abstract (plain language summary)

In health research, it is often not possible for all people affected by a topic to be directly involved in the research process. Instead, certain individuals speak or act on behalf of others – this is called ‘representation.’ This review will examine how researchers in Germany understand and apply the concept of representation in participatory health research. It will explore which individuals or groups are involved, how they are selected, and how their roles are described. By analyzing published studies, the review aims to provide an overview of current practices and identify areas for further development. The findings may support the future design of participatory research that does more justice to the perspectives of those affected.

**Supplementary Information:**

The online version contains supplementary material available at 10.1186/s40900-025-00736-w.

## Key message

This protocol outlines a scoping review that examines how representation is conceptualized and operationalized in participatory health research in Germany, mapping how different understandings of representation are reflected in study design and practice.

**Supplementary Information:**

The online version contains supplementary material available at 10.1186/s40900-025-00736-w.

## Background

Participatory health research (PHR) has developed significantly over the past decades, gaining increasing recognition as a way to integrate the perspectives of patients and communities into health research. In participatory research, individuals and groups affected by research can be involved in various processes, including the development of context-sensitive research questions and study designs [1], the practical implementation of research projects, or the dissemination of findings after the study is completed [2].

Early forms of participatory research can be traced back to the origins of action research in the mid-20th century [3]. However, its institutionalization in the field of health research did not begin until the late 20th century. A particularly influential initiative was INVOLVE, established in 1996 in the United Kingdom, which provided one of the first national frameworks for patient and public involvement in research [4]. Although INVOLVE was integrated into the NIHR Centre for Engagement and Dissemination in 2020, its foundational resources and principles remain influential. In the United States, the Patient-Centered Outcomes Research Institute (PCORI) was established through the Affordable Care Act in 2010. As of 2025, PCORI has funded more than 2,400 projects with a strong emphasis on patient involvement, making it one of the most significant drivers of participatory approaches in health research internationally [5]. Similarly, Canada’s Strategy for Patient-Oriented Research (SPOR), launched by the Canadian Institutes of Health Research (CIHR) in 2011, aims to integrate patients as active partners in research and has supported national networks and regional infrastructure to promote this goal [6].

In contrast, the German PHR landscape developed somewhat later and has not yet reached the same level of institutional embedding. A key step toward formalizing participatory approaches in health research was the establishment of the German Network for Participatory Health Research (PartNet) in 2007. PartNet promotes interdisciplinary exchange and methodological development and builds on traditions such as action research and community-based participatory research. These approaches view research as a co-productive process between those directly affected – such as patients, healthcare professionals, or civil society actors – and academic researchers [7, 8]. PHR in this tradition does not aim solely to generate knowledge, but also to promote health, well-being, and equity, while encouraging critical reflection on power dynamics [9].

A more recent initiative that further strengthens the institutional foundation for participatory and transdisciplinary research in Germany is the establishment of the Gesellschaft für transdisziplinäre und partizipative Forschung e.V. (GTPF, engl. *Society for Transdisciplinary and Participatory Research*). Founded in 2023, the GTPF supports researchers working at the intersection of science and society. It facilitates exchange among scholars in German-speaking countries and beyond, offers training opportunities for researchers at different career stages, and contributes to the further development of methods and teaching formats. In addition, the GTPF represents the interests of this research community in science policy debates and works to strengthen the role of participatory and transdisciplinary approaches in academia [10].

In recent years, German research funders have increasingly emphasized the relevance of PHR by explicitly requiring participatory approaches in their funding schemes [11–15]. In parallel to these policy developments, a broader narrative has emerged that frames PHR as a promising methodological framework capable of enhancing both the quality and the legitimacy of health research. It is often argued that participatory approaches increase the accountability and responsiveness of research to the needs of those affected [16], improve the efficiency of studies [17], and contribute to better implementation and uptake of results [18]. Moreover, PHR is frequently linked to democratic ideals, with the claim that it strengthens shared decision-making and mitigates democratic deficits in healthcare governance [17, 19, 20]. However, as Schickhardt et al. (2024) point out, the democratic value of participation is often assumed rather than conceptually clarified. Participation alone does not constitute democracy; rather, it requires an institutional framework that enables meaningful involvement and holds participants accountable [21]. Still, participation is often regarded as a fundamental source of legitimacy, particularly when it includes those directly affected by the outcomes – such as patients, relatives, caregivers, or community stakeholders – who can contribute experiential knowledge to improve the relevance and applicability of research.

At the same time, meaningful participation depends not only on whether individuals are involved, but also on whose perspectives are included. This raises critical questions about representation – particularly in relation to marginalized groups. According to Pitkin, representation involves “the making present of something which is nevertheless not literally present” [22]. In PHR, representation serves as a mechanism that enables selected individuals to speak or act on behalf of the broader communities they belong to, whose members cannot all be involved directly [23]. This warrants taking a look at these participatory practices using the framework of representation in order to better understand which voices are heard during these participatory processes and which voices are not, leading to unintended exclusions despite the participatory character of the research.

Concerns about selectivity and elitism in participatory approaches are well documented. Numerous studies point to patterns in which individuals with higher levels of education and socio-economic status are disproportionately represented in participatory processes – both in research and policy-making [18, 24–28]. This can undermine the epistemic diversity of research and risks reproducing existing forms of social inequality [29]. In response, representation is increasingly argued to be a potential quality criterion for participation although it would need further work on what constitutes adequate representation beforehand. Unlike output-oriented evaluations that focus on the measurable effectiveness of research, representation shifts attention to the input level – the procedural dimension – and serves as a proxy for the inclusiveness and legitimacy of participatory processes [21, 30, 31].

Despite its relevance, there is a lack of frameworks that allow for a systematic evaluation of representation in participatory health research. Most assessment efforts in the medical field focus on research outcomes rather than the quality or legitimacy of participatory processes [32]. This gap is particularly evident in the German context, where participatory approaches are increasingly promoted by research funders but still lack robust evaluative structures. Against this backdrop, the present scoping review explores how concepts of representation are operationalized in participatory health-related research in Germany.

Existing reviews of representation in participatory settings are scarce. One notable exception is a review by Lander et al. [33], which categorizes types of representation in public involvement activities in genomics. However, their analysis primarily focuses on recruitment logics and does not consider relational or normative understandings of representation. Our review builds on this earlier work by expanding the empirical scope to participatory health research in Germany and by introducing additional conceptual dimensions from political theory, such as representation as a responsive relationship and as an externally ascribed category.

## Relevant concepts

This review aims to determine which conceptualizations of representation are used in participatory settings in health research in Germany. We do this by using different conceptualizations of “representation” from political science and associated literature and examining their presence in the literature on participatory health research in Germany. We are guided by two concepts, which we use as deductive supercategories to classify operationalizations of representation in the literature. First, representation as a responsive relationship and, second, as externally ascribed representation (see Table 1). To account for additional forms of representation found in the literature alongside responsive representation and externally ascribed representation, the authors reserve the right to expand these deductive categories inductively during the screening process. This explicitly includes further subcategories of the two main conceptualizations mentioned.

Given its suitability for clarifying key concepts and definitions, we selected the scoping review format to consolidate existing knowledge and available evidence. This approach is considered a robust tool for analyzing research methodologies in particular domains and pinpointing crucial features or factors associated with given concepts [34].

**Table 1:** Relevant concepts

No	Concept	Definition
1	Participatory health research	Participatory health research (PHR) refers to a set of research approaches that actively involve individuals and groups affected by the research in one or more phases of the research process. These phases may include the formulation of research questions, the design and implementation of studies, data collection and analysis, as well as the interpretation and dissemination of results. The core idea is to shift research from a process done on or about people to one done with and by them [35]. Over time, multiple forms of PHR have emerged, including but not limited to Action Research or Community-Based Participatory Research (CBPR). These approaches are not always confined to the health sector but share a common emphasis on collaborative knowledge production and the empowerment of participants. The degree and nature of involvement can vary widely. Models such as Arnstein’s “Ladder of Citizen Participation” [36] and Charles and DeMaio’s framework [37] have been used to describe levels of participation, ranging from passive consultation to active partnership and citizen control. These models illustrate that not all participation is equal, and that power dynamics play a central role in determining the quality of participatory processes. Despite variations in terminology and implementation, participatory health research (PHR) is generally characterized by the following guiding principles: (1) recognition of participants as co-creators of knowledge; (2) some form of shared decision-making or mutual learning; and (3) a sensitivity to practical relevance or real-world applicability of research processes or outcomes. For the purposes of this scoping review, we apply a broad and inclusive understanding of participatory approaches. All studies that include at least one phase of research in which non-academic actors were actively involved in shaping the research process will be considered eligible, regardless of whether these studies pursue transformative goals (e.g., empowerment, social change). Related terms such as involvement, engagement, co-production, and participation are treated synonymously, unless the original study makes an explicit distinction.
2	Decision-making	In participatory research, decision-making refers to the joint processes through which involved actors – such as researchers, patients, community members, or service users – collaborate to shape key aspects of the research, including defining objectives, choosing methods, conducting analysis, and disseminating findings. It is grounded in shared authority, mutual learning, and the redistribution of power between academic and non-academic participants [36, 37].
3	Marginalized groups/people/communities	Marginalized groups are population groups who, due to social, economic, or political disadvantage, have limited access to decision-making processes. In the context of participatory research, these may include people with disabilities, individuals experiencing drug use and homelessness [38], as well as migrants, refugees, and ethnic minorities [39]. While participatory approaches are often normatively associated with the inclusion and empowerment of such groups, many studies do not explicitly focus on them. Nonetheless, there are participatory projects in which members of marginalized communities are actively involved as co-researchers, co-analysts, or even as designers and producers of their own research [40].
4	Stakeholder	Stakeholders are individuals or groups who can affect or are affected by the achievement of an organization’s objectives [41]. In participatory research, stakeholders are understood as individuals or groups who have an interest in the research topic, are affected by it, or have the potential to influence its processes or outcomes due to their roles, experiences, or positions [42]. These may include patients and their social networks, healthcare professionals, policymakers, or funders.
5	Representation	Representation can be understood as the act of speaking or acting on behalf of others who are not physically present, thereby making their interests, identities, or values visible in decision-making processes [22, 43]. The concept originates from political and legal theory and appears in various conceptualizations. In the context of this scoping review, we distinguish between “representation as a responsive relationship” and “representation as externally ascribed representation” (see rows 6 to 7.2).
6	Representation as a responsive relationship	In participatory research, representation can be understood as a responsive relationship between participants and those not directly involved in the research setting but affected by its outcomes. This relationship is shaped by different modes of representation, which can be distinguished in three types (see row 6.1 to 6.3).
6.1	Representation based on formal and informal mechanisms of authorization and accountability	Representation can be based on formal or informal mechanisms of authorization and accountability. This type of representation is based on a dynamic, responsive relationship, in which those represented authorize individuals to speak or act on their behalf and can hold them accountable for their actions [44, 45, 30]. Authorization mechanisms describe the processes by which “representatives are selected or directed by those they claim to represent” (46). Accountability mechanisms, on the other hand, are those that “explain and justify the behavior of the representatives to those they represent” (46). Overall, these can take a wide range of shapes and forms, with formal elections certainly being the most prominent example. Further, such mechanisms can include petitions, protests and many other informal ways of authorizing representatives and holding them accountable [47, 44, 46].
6.2	Representation based on shared identity, specific knowledge and skills or shared experiences between representatives and those represented	Here, representation is grounded in descriptive or experiential similarity between representatives and those represented. This may include shared social characteristics (e.g. gender, ethnicity), common experiences (e.g. illness), or specific expertise that enables representatives to interpret and convey the concerns of others [48–50, 44, 30, 51].
6.3	Trustee and delegate representation	Trustee and delegate representation refers to two distinct modes of representing others. In the delegate model, representatives act strictly in accordance with the expressed preferences of those they represent, functioning as direct transmitters of their views. In contrast, the trustee model allows representatives to use their own judgment to interpret and act in the presumed best interest of the represented. This typology highlights different expectations regarding the autonomy and responsibility of representatives [22, 43, 52].
7	Externally ascribed representation	Externally ascribed representation refers to forms of representation that do not primarily center on the relationship between participants in a research setting and those not present. Instead, it highlights the sampling and recruitment logics applied by researchers to construct a study population. These forms of representation are assigned from the outside and are shaped by methodological decisions rather than participatory claims. We distinguish two types (see rows 7.1 and 7.2).
7.1	Statistical representativeness	In the context of participatory research, statistical representativeness refers to efforts to compose groups of participating stakeholders – such as patient representatives, advisory board members, or co-researchers – in a way that reflects the socio-demographic characteristics of a larger reference population. This approach aims to ensure that the perspectives included in the participatory process are broadly reflective of the population affected by the research topic, based on categories such as age, gender, income, or ethnicity.
7.2	Qualitative sampling logics	Qualitative sampling logics include non-probabilistic strategies such as theoretical sampling, purposive sampling, snowball sampling, or convenience sampling. These approaches aim to capture diverse perspectives, rich information, or theoretically relevant cases rather than statistical generalizability. Representation here is based on the intentional inclusion of certain types of knowledge or experience rather than numeric proportion [53, 33].

## Methods

The scoping review will be conducted in accordance with the JBI methodology for scoping reviews [54, 55] and reported following the Preferred Reporting Items for Systematic Reviews and Meta-Analyses extension for scoping reviews (PRISMA-ScR) [56]. The following section outlines the different stages of the review process. Table 2 outlines the eight key stages of the scoping review methodology.

**Table 2:** Overview of the methodological stages of the scoping review

Stage	Title
**1**	Identification of the research question
**2**	Creation of a search strategy
**3**	Application of the search strategy
**4**	Quality assurance via pilot testing
**5**	Screening of documents
**6**	Data extraction
**7**	Data analysis and presentation
**8**	Consultation and dissemination

### Stage 1: identification of the research question

The research question was developed in an iterative process guided by preliminary literature screening, team discussions, and the conceptual framing of participation and representation. The objective of the scoping review is to identify and analyze how representation is conceptualized and operationalized in participatory health-related research conducted in Germany. This overarching research question was further refined through consultation of key literature and existing conceptual frameworks, especially from political science, public health, and participatory methodology.

### Stage 2: creation of a search strategy

The identification of the search strategy closely follows the suggestions made by Aromataris and Munn [54]. At the protocol level, a limited search of two related databases (Medline via Pubmed, Web of Science Core Collection) was undertaken to identify articles on the topic. The terms contained in the titles and abstracts of these articles as well as the index terms used to describe the articles were used to develop a search strategy for the electronic databases (see Appendix I for the search strategy for Pubmed, which will be adapted for the other databases according to their guidelines).

### Stage 3: application of the search strategy

In the main review phase, a comprehensive search will be conducted using the keywords and index terms identified during the preliminary search. The search will be applied across multiple electronic databases relevant to the interdisciplinary scope of the review, including CINAHL, Medline via PubMed, PsycINFO, and Scopus. These databases were selected to ensure coverage of the fields most relevant to participatory health research, namely medicine, public health, psychology, nursing, and the social sciences.

The search strategy will be tailored to the indexing systems and search functionalities of each database to ensure consistency and comprehensiveness. In addition to database searches, a manual screening of the document archive maintained by PartNet will be carried out, as it represents a key repository of participatory health research in the German-speaking context [57]. Furthermore, reference lists of all included articles will be scanned to identify additional relevant literature. This step is intended to enhance the sensitivity of the search strategy and to minimize the risk of missing key publications [54].

### Stage 4: quality assurance via pilot testing

To enhance the reliability and consistency of the study selection process, a pilot screening will be conducted independently by two reviewers. For this purpose, 25 records (titles and abstracts) will be randomly selected and assessed based on the predefined eligibility criteria (see Table 3). In cases where abstracts are missing, the full texts will be screened. The results of the two reviewers will then be compared to determine the level of agreement. Any discrepancies will be discussed in order to refine the eligibility criteria if necessary.

The main screening process will commence only if a minimum agreement rate of 75% between reviewers is reached. A similar procedure will be applied to the full-text screening: 25 full texts will be randomly selected and evaluated independently by both reviewers. Again, a 75% agreement rate will be required before proceeding to the full-text screening phase of the review. This two-tiered pilot testing is designed to ensure clarity, consistency, and transparency in the application of inclusion and exclusion criteria.

### Stage 5: screening of documents

Following successful pilot testing, the main screening process will be conducted in two steps. First, two independent reviewers will screen all identified titles and abstracts to assess their relevance based on the predefined inclusion criteria. Records that do not meet these criteria – particularly those that do not clearly indicate a participatory research context – will be excluded at this stage. In the second step, the full texts of all remaining records will be independently reviewed by both researchers. Reasons for exclusion at the full-text level will be documented in detail. Any disagreements during either stage will be resolved through discussion; if no consensus can be reached, a third reviewer will be consulted.

To support and document the screening process, the JBI System for the Unified Management, Assessment and Review of Information (JBI SUMARI) will be used. This tool facilitates the systematic identification, selection, and documentation of relevant studies in accordance with the JBI methodology. The overall selection process will be visualized using a PRISMA-ScR flow diagram, and separate appendices will provide detailed listings of included and excluded full-text sources, along with the respective reasons for exclusion.

### Stage 6: data extraction

Data will be extracted independently by two reviewers using a standardized charting form. A preliminary version of the charting form was developed during the protocol stage, based on the JBI template and tailored to the specific objectives of this review [54]. The draft version of this table is provided in Appendix II. It captures key information including bibliographic details, study design, participatory approach, conceptualizations of representation, sampling strategies, and mechanisms of authorization and accountability.

Prior to full application, the charting form will be pilot-tested on five randomly selected studies. Any discrepancies between the reviewers will be discussed and may lead to further refinement of the table. Data extraction and management will be supported using the JBI SUMARI platform to ensure transparency, consistency, and traceability throughout the process.

### Stage 7: data analysis and presentation

The primary aim of the analysis is to identify and categorize the conceptualizations of representation used in participatory health research in Germany. To this end, all included documents will be examined using qualitative content analysis, following both deductive and inductive approaches. Deductive coding will be guided by the two overarching conceptual categories defined in the protocol—representation as a responsive relationship and externally ascribed representation (see Table 1). In addition, inductive coding will allow for the identification of further subcategories or alternative forms of representation that emerge from the material.

The qualitative content analysis will be conducted using the software MAXQDA, which supports systematic coding, category development, and the visualization of relationships between identified concepts. In addition to conceptual dimensions, the analysis will explore which types of actors – such as patients, relatives, or healthcare professionals – are involved as representatives or are being represented. This differentiation allows for a more nuanced understanding of how representation is constructed and attributed across stakeholder groups, and whether specific conceptualizations of representation are more commonly associated with certain types of participants. If applicable, the analysis will be expanded by capturing descriptive characteristics such as research discipline, type of publication, and year of publication, in order to identify possible patterns or trends in the operationalization of representation over time or across disciplinary contexts. The findings will be presented in accordance with the PRISMA-ScR Statement [56]. Results will be visualized using tables and histograms to show the frequency and distribution of representation-related concepts. Where applicable, figures or charts will be used to illustrate temporal or disciplinary trends and explore potential correlations between different conceptualizations of representation.

### Stage 8: consultation and dissemination

To ensure the conceptual robustness and practical relevance of the review findings, a consultation phase with experienced individuals from participatory health research will be integrated into the review process [58]. An expert group of approximately eight participants will be convened, including patient partners and health professionals, patient organization representatives and researchers with a background in co-productive or participatory methodologies as well as political science. The group will be involved in reviewing preliminary findings and discussing their applicability, clarity, and potential implications for participatory practice and policy. Feedback gathered through this exchange will help refine the interpretation of results and ensure that the conceptualizations of representation identified in the review resonate with real-world participatory contexts. The nature and scope of involvement will be documented using the GRIPP2 short form checklist [59]. The findings of this scoping review will be disseminated through peer-reviewed publications, conference presentations, and knowledge exchange formats targeting researchers, practitioners, and patient communities.

### Inclusion and exclusion criteria

The selection of articles is based on a set of inclusion and exclusion criteria, which are outlined in Table 3. This review will include those scientific articles making explicit use of PHR in Germany. Eligible studies must be led by or conducted in collaboration with a German research institution and must have implemented a participatory approach in at least one phase of the research process (e.g., planning, recruitment, data collection, analysis, or dissemination), either in Germany or in an online context.

This review focuses on the German research context for several reasons. Participatory health research is highly context-dependent, and its conceptual and practical implementation is shaped by country-specific factors such as research culture, healthcare structures, and institutional support. An in-depth focus on Germany allows for a more coherent and contextually grounded analysis of how representation is understood and applied. Moreover, international terminology and practices in PHR vary widely, which complicates comparative analysis unless national contexts are first systematically examined. Finally, while participatory approaches are increasingly discussed at the international level, the conceptual landscape in Germany has not yet been mapped in detail. This review addresses this gap and lays the groundwork for future comparative studies.

To reflect the development of participatory health research in Germany, only studies published between 1990 and 2025 will be included, with the final search conducted in June 2025 defining the cut-off. All databases will be searched up to this point. Due to the incomplete coverage of 2025, any temporal comparisons will be adjusted accordingly. This time frame may be extended, if necessary, based on findings during the screening process. Only studies conducted in German or English will be considered.

No eligibility criteria are set regarding the composition of participants in the research settings. However, studies must be situated within the health sciences or adopt an interdisciplinary approach with a clear health-related component. Health research is defined in the broadest possible sense and includes, but is not limited to, biomedical and clinical research, epidemiology, health economics, health systems and services research, environmental health, and behavioral and social science health research.

**Table 3:** Inclusion and exclusion criteria for the scoping review

Criterion	Inclusion	Exclusion
**Study type**	Peer-reviewed scientific articles reporting on primary, empirical research	Theoretical or conceptual papers; commentaries; editorials; gray literature; conference abstracts
**Language**	German or English	All other languages
**Publication date**	1990–2025 (modifiable depending on review findings)	Before 1990 (unless scope is adapted)
**Geographical focus**	Studies conducted in Germany or online, with lead or collaboration from a German institution	Studies without any connection to German research institutions
**Participatory approach**	Explicit use of participatory health research in at least one research phase	No reference to participatory approaches or only rhetorical/ informal mentions
**Disciplinary focus**	Health sciences or interdisciplinary studies with a clear health-related component	Studies not addressing health, disease, healthcare, or well-being
**Accessibility**	Freely accessible online or accessible through institutional access	Not available due to paywalls or missing access
**Data sources**	Full-text articles including appendices (if relevant content is present)	Incomplete publications or abstracts without sufficient data
**Level of participation**	Any degree or form of participation, as long as it is explicitly described	Studies where participation is claimed but not operationalized
**Type of research phase involved**	Participation in any research phase (e.g., planning, recruitment, data collection, analysis)	Studies where no participatory role in the research process can be identified

## Discussion

This scoping review addresses a significant gap by systematically mapping how representation is conceptualized and operationalized in participatory health research in Germany. A key strength of the review lies in its comprehensive and structured methodological approach, which is grounded in established frameworks such as the JBI methodology and the PRISMA-ScR checklist. By using both deductive and inductive coding in qualitative content analysis and incorporating stakeholder consultation, the review ensures conceptual depth, methodological rigor, and practical relevance. Although the review only includes empirical studies, its analysis is guided by theoretical frameworks from political theory, allowing for a nuanced examination of how representation is addressed in practice.

At the same time, the review is subject to certain limitations. First, the geographical scope is restricted to Germany, which may limit the transferability of findings to other countries. However, this focused perspective allows for detailed contextual analysis and provides a foundation for future comparative work. Second, the exclusive inclusion of peer-reviewed literature may result in the omission of relevant insights from grey literature or ongoing participatory projects. Finally, as the review synthesizes published material, it relies on how well studies report on representation, which may vary in depth and clarity.

Despite these limitations, the review offers a valuable contribution to current debates on the legitimacy and inclusivity of participatory health research and may inform future evaluation frameworks by highlighting how representation is conceptualized and implemented in practice. Although the scope of this review is limited to the German context, the conceptual frameworks and operationalizations of representation identified may be of relevance to other countries with comparable institutional and cultural settings, such as Austria or other German-speaking regions. The findings can serve as a reference point for future cross-national comparisons and inform the design of participatory processes beyond Germany.

## Electronic supplementary material

Below is the link to the electronic supplementary material.

Supplementary Material 1

Supplementary Material 2

## Data Availability

No datasets were generated or analysed during the current study.

## Abbreviations

CIHR: Canadian Institutes of Health Research
GTPF: Gesellschaft für transdisziplinäre und partizipative Forschung e.V
GRIPP2: Guidance for Reporting Involvement of Patients and the Public 2
NIHR: National Institute for Health and Care Research
PartNet: German Network for Participatory Health Research
PCORI: Patient–Centered Outcomes Research Institute
PHR: Participatory Health Research
PRISMA-ScR: Preferred Reporting Items for Systematic Reviews and Meta–Analyses Extension for Scoping Reviews
SPOR: Strategy for Patient–Oriented Research
