# Supplementary material for: The role of representation in participatory settings of health research in Germany: protocol for a scoping review
Source: Res Involv Engagem. 2025 Jul 3;11:75. doi: 10.1186/s40900-025-00736-w (PMC12226853; doi:10.1186/s40900-025-00736-w)
Supplement: Supplementary file 1 — Supplementary Material 1 [file 40900_2025_736_MOESM1_ESM.docx]

**Appendix I: Exemplary search strategy for Pubmed by Medline**

| CONCEPT | CONTEXT (LOCATION) |
| --- | --- |
| („action research“[Title/Abstract] OR „advoca* consultation“[Title/Abstract] OR „advoca* contribution“[Title/Abstract] OR „advoca* engagement“[Title/Abstract] OR „advoca* involvement“[Title/Abstract] OR „advoca* participation“[Title/Abstract] OR „advocate-driven“[Title/Abstract] OR „advocate-led“[Title/Abstract] OR „Aktionsforschung“[Title/Abstract] OR „beteilig*“[Title/Abstract] OR „Bürgerbeteiligung“[Title/Abstract] OR „Bürger-Beteiligung“[Title/Abstract] OR „Bürgerinvolvierung“[Title/Abstract] OR „Bürger-Involvierung“[Title/Abstract] OR „Bürgerwissenschaft*“[Title/Abstract] OR „caregiver* consultation“[Title/Abstract] OR „caregiver* contribution“[Title/Abstract] OR „caregiver* engagement“[Title/Abstract] OR „caregiver* involvement“[Title/Abstract] OR „caregiver* participation“[Title/Abstract] OR „caregiver-driven“[Title/Abstract] OR „caregiver-led“[Title/Abstract] OR „citizen consultation“[Title/Abstract] OR „citizen contribution“[Title/Abstract] OR „citizen engagement“[Title/Abstract] OR „citizen involvement“[Title/Abstract] OR „citizen participation“[Title/Abstract] OR „citizen science“[Title/Abstract] OR „citizen-driven“[Title/Abstract] OR „citizen-led“[Title/Abstract] OR „client* consultation“[Title/Abstract] OR „client* contribution“[Title/Abstract] OR „client* engagement“[Title/Abstract] OR „client* involvement“[Title/Abstract] OR „client* participation“[Title/Abstract] OR „co-design“[Title/Abstract] OR „community consultation“[Title/Abstract] OR „community contribution“[Title/Abstract] OR „community engagement“[Title/Abstract] OR „community involvement“[Title/Abstract] OR „community participation“[Title/Abstract] OR „community partner“[Title/Abstract] OR „community research“[Title/Abstract] OR „community-driven“[Title/Abstract] OR „Community-Engagement“[Title/Abstract] OR „community-led“[Title/Abstract] OR „consumer consultation“[Title/Abstract] OR „consumer contribution“[Title/Abstract] OR „consumer engagement“[Title/Abstract] OR „consumer involvement“[Title/Abstract] OR „consumer participation“[Title/Abstract] OR „consumer-driven“[Title/Abstract] OR „consumer-led“[Title/Abstract] OR „coproduc*“[Title/Abstract] OR „co-produc*“[Title/Abstract] OR „co-research“[Title/Abstract] OR „Ko-Forsch*“[Title/Abstract] OR „Koproduktion“[Title/Abstract] OR „Ko-Produktion“[Title/Abstract] OR „Laienbeteiligung“[Title/Abstract] OR „lay consultation“[Title/Abstract] OR „lay contribution“[Title/Abstract] OR „lay engagement“[Title/Abstract] OR „lay engagement“[Title/Abstract] OR „lay involvement“[Title/Abstract] OR „lay involvement“[Title/Abstract] OR „lay participation“[Title/Abstract] OR „lay-driven“[Title/Abstract] OR „lay-led“[Title/Abstract] OR „Öffentlichkeitsbeteiligung“[Title/Abstract] OR „parent* consultation“[Title/Abstract] OR „parent* contribution“[Title/Abstract] OR „parent* engagement“[Title/Abstract] OR „parent* involvement“[Title/Abstract] OR „parent* participation“[Title/Abstract] OR „participatory design“[Title/Abstract] OR „participatory research“[Title/Abstract] OR „partizipa*“[Title/Abstract] OR „patient consultation“[Title/Abstract] OR „patient contribution“[Title/Abstract] OR „patient engagement“[Title/Abstract] OR „patient involvement“[Title/Abstract] OR „patient participation“[Title/Abstract] OR „patient partner*“[Title/Abstract] OR „patient-centered research“[Title/Abstract] OR „patient-centred research“[Title/Abstract] OR „patient-driven“[Title/Abstract] OR „patient-driven“[Title/Abstract] OR „Patientenbeteiligung“[Title/Abstract] OR „Patienteninvolvierung“[Title/Abstract] OR „Patienten-Involvierung“[Title/Abstract] OR „Patientenpartner“[Title/Abstract] OR „patient-led“[Title/Abstract] OR „patient-led“[Title/Abstract] OR „peer research“[Title/Abstract] OR „Peer-Forschung“[Title/Abstract] OR „peer-led research“[Title/Abstract] OR „PPI“[Title/Abstract] OR „public consultation“[Title/Abstract] OR „public contribution“[Title/Abstract] OR „public engagement“[Title/Abstract] OR „public involvement“[Title/Abstract] OR „public participation“[Title/Abstract] OR „public-driven“[Title/Abstract] OR „public-led“[Title/Abstract] OR „relative* consultation“[Title/Abstract] OR „relative* contribution“[Title/Abstract] OR „relative* engagement“[Title/Abstract] OR „relative* involvement“[Title/Abstract] OR „relative* participation“[Title/Abstract] OR „stakeholder consultation“[Title/Abstract] OR „stakeholder contribution“[Title/Abstract] OR „stakeholder engagement“[Title/Abstract] OR „stakeholder involvement“[Title/Abstract] OR „stakeholder participation“[Title/Abstract] OR „Stakeholder-Beteiligung“[Title/Abstract] OR „stakeholder-driven“[Title/Abstract] OR „stakeholder-led“[Title/Abstract] OR „surrogate consultation“[Title/Abstract] OR „surrogate contribution“[Title/Abstract] OR „surrogate engagement“[Title/Abstract] OR „surrogate involvement“[Title/Abstract] OR „surrogate participation“[Title/Abstract] OR „surrogate-driven“[Title/Abstract] OR „surrogate-led“[Title/Abstract] OR „user consultation“[Title/Abstract] OR „user contribution“[Title/Abstract] OR „user engagement“[Title/Abstract] OR „user involvement“[Title/Abstract] OR „user participation“[Title/Abstract] OR „user-driven“[Title/Abstract] OR „user-led“[Title/Abstract]) | (“Baden-Württemberg“[Title/Abstract] OR „baden-württembergisch*“[Title/Abstract] OR „bayerisch*“[Title/Abstract] OR „Bayern“[Title/Abstract] OR „Berlin“[Title/Abstract] OR „berlinerisch*“[Title/Abstract] OR „Brandenburg“[Title/Abstract] OR „brandenburgisch*“[Title/Abstract] OR „Bremen“[Title/Abstract] OR „bremisch*“[Title/Abstract] OR „deutsch*“[Title/Abstract] OR „German*“[Title/Abstract] OR „Hamburg“[Title/Abstract] OR „hamburgerisch*“[Title/Abstract] OR „Hessen“[Title/Abstract] OR „hessisch*“[Title/Abstract] OR „Mecklenburg-Vorpommern“[Title/Abstract] OR „mecklenburg-vorpommersch*“[Title/Abstract] OR „Niedersachsen“[Title/Abstract] OR „niedersächsisch*“[Title/Abstract] OR „Nordrhein-Westfalen“[Title/Abstract] OR „nordrhein-westfälisch*“[Title/Abstract] OR „Rheinland-Pfalz“[Title/Abstract] OR „rheinland-pfälzisch*“[Title/Abstract] OR „Saarland“[Title/Abstract] OR „saarländisch*“[Title/Abstract] OR „Sachsen“[Title/Abstract] OR „Sachsen-Anhalt“[Title/Abstract] OR „sachsen-anhältisch*“[Title/Abstract] OR „sächsisch*“[Title/Abstract] OR „Schleswig-Holstein“[Title/Abstract] OR „schleswig-holsteinisch*“[Title/Abstract] OR „Thüringen“[Title/Abstract] OR „thüringisch*“[Title/Abstract]) |
