# Supplementary material for: The role of representation in participatory settings of health research in Germany: protocol for a scoping review
Source: Res Involv Engagem. 2025 Jul 3;11:75. doi: 10.1186/s40900-025-00736-w (PMC12226853; doi:10.1186/s40900-025-00736-w)
Supplement: Supplementary file 2 — Supplementary Material 2 [file 40900_2025_736_MOESM2_ESM.docx]

**Appendix II: Adjusted draft charting form according to JBI methodology for scoping reviews**

| 1. **Scoping review details** | |
| --- | --- |
| - 1. Scoping Review title |  |
| - 1. Review objective/s |  |
| - 1. Review question/s |  |
| 1. **Evidence source details and characteristics** | |
| - 1. Author/s |  |
| - 1. Title of publication |  |
| - 1. Date of publication |  |
| - 1. Journal of publication |  |
| - 1. Volume and issue of publication |  |
| - 1. Aims/purpose of the publication/study |  |
| - 1. Type of study |  |
| - 1. Sociodemographic data of study  participants |  |
| - 1. Institution/s conducting the study |  |
| - 1. Funding Institution/s |  |
| 1. **Information about participatory approach** | |
| - 1. Sociodemographic data of participating individuals (actively involved as opposed to 2.8.) |  |
| - 1. Stage of research process using  participatory methods |  |
| - 1. Participatory methods employed |  |
| 1. **Details/results extracted from source of evidence** | |
| - 1. Concept of Representation |  |
| - - 1. Mechanisms of authorization |  |
| - - 1. Mechanisms of accountability |  |
| - - 1. Representation based on shared identity, specific knowledge and skills or shared experiences between representatives and those represented |  |
| - - 1. Trustee representation |  |
| - - 1. Delegate representation |  |
| - - 1. Statistical representativeness |  |
| - - 1. Qualitative Sampling Logics |  |
| - - 1. Other forms of representation |  |
